# Self-Supervised OCT Representation Learning with Local Dimensionality Regularization for Automated Retinal Disease Diagnosis

**DOI:** 10.3390/s26175338

**Published:** 2026-08-23

**Authors:** Xiangge Sun, Wenrui Lin, Chenao Yuan, Jun Xu, Yuemei Luo

**Affiliations:** Jiangsu Key Laboratory of Intelligent Medical Image Computing (IMIC), School of Artificial Intelligence/School of Future Technology, Nanjing University of Information Science and Technology, Nanjing 210044, China; 202383460050@nuist.edu.cn (X.S.); lwrnuist@163.com (W.L.); cayuan@nuist.edu.cn (C.Y.); jxu@nuist.edu.cn (J.X.)

**Keywords:** retinal disease diagnosis, OCT image classification, self-supervised representation learning, local intrinsic dimensionality, dimensionality regularization

## Abstract

Optical coherence tomography (OCT) is a high-resolution and non-contact optical imaging and sensing modality that provides depth-resolved cross-sectional visualization of retinal microstructures. It plays an important role in the assessment of retinal diseases, including age-related macular degeneration (AMD) and diabetic macular edema (DME). However, automated OCT image classification commonly relies on fully supervised models that require large-scale expert annotations, which are costly and time-consuming because of the complex layered anatomy and subtle pathological patterns present in retinal OCT images. To reduce annotation dependence, this study proposes a self-supervised representation learning framework with local dimensionality regularization for retinal OCT image classification. The proposed method estimates the local intrinsic dimensionality of learned representations and incorporates it into an asymptotic Fisher-Rao regularization objective to mitigate local dimensional degeneration and preserve fine-grained structural information. Logarithmic scaling and geometric averaging are further introduced to reduce sensitivity to outliers and improve optimization stability. Experiments on three independent OCT datasets achieved classification accuracies of 94.35%, 92.48%, and 92.56%, respectively, demonstrating competitive performance compared with mainstream self-supervised methods. These results demonstrate that explicitly modeling local feature geometry can improve the discrimination of sensor-acquired OCT images while reducing reliance on manual annotations, providing an effective approach for intelligent analysis of biomedical optical imaging data.

## 1. Introduction

Optical coherence tomography (OCT) is a non-contact optical imaging and sensing modality that uses low-coherence interferometry to provide high-resolution and depth-resolved visualization of retinal structures for disease assessment [1]. Retinal OCT images reveal layered anatomical structures and pathological changes associated with age-related macular degeneration (AMD), diabetic macular edema (DME), choroidal neovascularization (CNV), and drusen [2,3]. Nevertheless, subtle variations in retinal thickness, layer continuity, optical reflectivity, and fluid accumulation can be difficult and time-consuming to identify through manual inspection. The rich structural information provided by OCT therefore offers a valuable basis for computational imaging, intelligent interpretation of sensor-acquired retinal data, and automated retinal disease classification, motivating the development of deep learning-based OCT image analysis methods [4,5].

Deep learning has substantially advanced retinal OCT analysis by enabling models to learn discriminative representations directly from image data. Existing studies have demonstrated promising performance in image reconstruction, lesion analysis, disease classification, and clinical decision support. For example, hybrid deep learning frameworks and multi-scale residual convolutional networks have achieved competitive performance in OCT-based retinal disease classification by exploiting discriminative structural features at different spatial scales [5,6]. However, most current approaches are developed under fully supervised settings and depend on large quantities of accurately annotated images. Producing reliable OCT annotations requires specialized ophthalmic expertise and careful interpretation of subtle pathological patterns, making large-scale labeling expensive and labor-intensive. The limited availability of high-quality annotations consequently remains an important obstacle to the development of scalable medical image analysis systems [7].

In contrast, large volumes of unlabeled OCT images are routinely accumulated during clinical imaging procedures. Self-supervised learning provides a practical means of exploiting these data by constructing supervisory signals directly from the images and learning transferable representations without extensive manual annotation [8,9]. Previous studies have shown that self-supervised pre-training can improve OCT representation learning and provide effective initialization for downstream retinal image classification [10,11,12]. This learning paradigm is particularly suitable for OCT analysis because it can exploit the repeated anatomical organization and structural regularities present in unlabeled retinal scans.

Despite this potential, general-purpose self-supervised learning objectives are not specifically designed for the local structural characteristics of retinal OCT images. Many existing methods emphasize global view consistency, reconstruction, redundancy reduction, latent prediction, or autoregressive modeling. Although these strategies can produce effective global representations, diagnostically relevant OCT information is often associated with localized changes in retinal layers and lesion morphology. Representations that preserve global image characteristics may still exhibit local dimensional degeneration, in which neighboring samples become excessively concentrated within a restricted feature subspace. Such local concentration can reduce feature diversity and make it difficult to distinguish images with similar overall appearance but different localized pathological patterns.

Local intrinsic dimensionality (LID) provides a sample-level measure of the effective dimensional complexity within a feature neighborhood. Unlike global dimensionality measures, LID characterizes the rate at which local probability mass expands around an individual representation and can therefore reveal locally redundant or dimensionally degenerate feature structures. Local intrinsic dimensionality regularization has recently been investigated in general self-supervised representation learning [13]. However, its effectiveness for retinal OCT data, where layered anatomy, subtle lesions, and device-dependent appearance variations jointly influence the learned feature space, remains insufficiently studied.

Although the original LDReg framework [13] introduced local dimensionality regularization for general self-supervised representation learning, its primary objective was to improve representation learning on general visual data rather than retinal OCT analysis. The original framework does not explicitly consider the layered anatomical characteristics of retinal OCT images, the subtle local pathological variations associated with retinal diseases, or the OCT-specific data augmentation and evaluation protocols required for medical image analysis. Therefore, directly applying the original LDReg framework to retinal OCT images cannot fully exploit the intrinsic geometric characteristics of retinal representations.

Accordingly, this study develops an OCT-oriented self-supervised representation learning framework with local intrinsic dimensionality regularization. Rather than proposing a new local dimensionality regularization theory, the present work reformulates the objective of local intrinsic dimensionality regularization toward preserving clinically meaningful retinal anatomical and pathological variations during self-supervised representation learning. Specifically, local intrinsic dimensionality is estimated from neighborhood distance distributions of retinal feature representations and incorporated into an asymptotic Fisher-Rao regularization objective. By jointly optimizing the self-supervised learning objective and the proposed local geometric constraint, the framework alleviates local dimensional degeneration while maintaining discriminative retinal structural representations.

Compared with the original LDReg framework [13], the present study differs in several important aspects. First, while the original LDReg framework [13] investigates local dimensionality regularization as a generic geometric constraint for self-supervised representation learning, the present work reformulates the optimization objective toward preserving diagnostically meaningful retinal anatomical and pathological variations. Second, unlike the original LDReg framework [13], which treats local geometry as a generic property of the learned feature manifold, our framework explicitly models local geometric structures corresponding to retinal tissue organization and subtle lesion morphology, enabling local intrinsic dimensionality regularization to better preserve clinically discriminative representations. Third, the proposed framework integrates local intrinsic dimensionality regularization into a complete retinal OCT self-supervised learning pipeline, jointly optimizing representation discrimination and local geometric preservation throughout the entire pre-training process. Finally, extensive experiments on three public retinal OCT datasets under linear and semi-supervised evaluation protocols demonstrate the effectiveness of the proposed framework for retinal OCT representation learning.

In this study, we specifically focus on two-dimensional structural retinal OCT images (B-scans) for self-supervised representation learning and automated retinal disease classification.

The main contributions of this study are summarized as follows:

1. We develop an OCT-oriented self-supervised representation learning framework that reformulates local intrinsic dimensionality regularization toward preserving clinically meaningful retinal anatomical and pathological variations by incorporating neighborhood-level dimensional information into retinal OCT feature learning, thereby reducing local dimensional degeneration while maintaining discriminative retinal representations.
2. We integrate local intrinsic dimensionality estimation with the asymptotic Fisher-Rao regularization into the self-supervised optimization process, enabling effective local geometric regularization without explicitly computing the Fisher information matrix or optimizing geodesic paths.
3. We conduct comprehensive evaluations on three public retinal OCT datasets under both linear evaluation and limited-label semi-supervised protocols. The quantitative metrics, ROC and PR curves, and confusion matrix analyses demonstrate the effectiveness of local dimensionality regularization for retinal OCT representation learning.

## 2. Related Works

### 2.1. Deep Learning for Retinal OCT Analysis

Deep learning has been widely applied to retinal OCT analysis, including image reconstruction, retinal layer segmentation, lesion detection, and disease classification. Kermany et al. developed a supervised convolutional neural network to classify OCT images into normal, choroidal neovascularization (CNV), diabetic macular edema (DME), and drusen categories, establishing a widely used benchmark for automated retinal disease classification [3]. De Fauw et al. proposed a clinically oriented deep learning system that combined retinal tissue segmentation with disease assessment and referral recommendation, demonstrating the potential of deep neural networks for interpreting large-scale clinical OCT data [14].

Transformer-based architectures have subsequently been introduced to capture long-range dependencies and hierarchical structural information in OCT images. For example, OCTFormer integrates transformer-based modeling with hierarchical feature extraction for retinal OCT classification across multiple datasets [15]. These studies demonstrate that supervised deep learning can effectively extract discriminative anatomical and pathological information from retinal OCT images.

Despite their promising performance, supervised approaches generally require large quantities of accurately annotated data. Producing reliable OCT annotations requires specialized ophthalmic expertise and careful interpretation of subtle variations in retinal layers, reflectivity, and lesion morphology. This dependence on expert labels limits the scalability of supervised models and motivates the development of learning strategies that can exploit unlabeled OCT images.

### 2.2. Unsupervised and Semi-Supervised Learning for Retinal OCT Analysis

Unsupervised learning has been investigated in retinal OCT analysis to reduce dependence on detailed annotations. Seeböck et al. combined autoencoder-based feature learning with a support vector machine to model normal retinal appearance and identify abnormal regions in OCT images [16]. Features learned from healthy samples were used to characterize normal tissue distributions, allowing deviations to be detected as potential abnormalities. The detected regions were subsequently clustered to identify recurrent patterns and examine their associations with retinal diseases.

Semi-supervised learning combines a limited number of labeled samples with a larger collection of unlabeled images. Twin self-supervision-based semi-supervised learning has been investigated for retinal anomaly classification under limited-annotation conditions [17], showing that label information and consistency signals derived from unlabeled data can jointly improve feature discrimination. Hu et al. further proposed a contrastive graph regularization framework for OCT disease identification with limited labeled data [18]. Their method exploits neighborhood relationships to refine pseudo-labels and regularize feature learning, thereby improving robustness to unreliable pseudo-labels.

More recently, Li et al. proposed a class-aware contrastive semi-supervised framework for retinal disease identification from OCT images [19]. The method integrates class-aware contrastive learning with FixMatch and incorporates pseudo-label selection and confidence-based prediction reweighting to reduce confirmation bias. A supervised contrastive objective is further used to model class-level relationships and improve the discrimination of retinal diseases with similar visual characteristics.

Although these unsupervised and semi-supervised approaches reduce dependence on fully annotated OCT datasets, conventional unsupervised methods often separate feature extraction from downstream classification, whereas semi-supervised methods still require labeled samples and may remain sensitive to pseudo-label quality. Self-supervised learning adopts a different paradigm by constructing supervisory signals directly from unlabeled data and transferring the learned representations to downstream tasks.

### 2.3. General Self-Supervised Visual Representation Learning

Self-supervised visual representation learning can be broadly categorized according to the supervisory objective used during pre-training. Contrastive methods learn transformation-invariant representations by attracting positive image pairs while separating negative samples. SimCLR generates two augmented views of each image and applies a contrastive objective to learn discriminative representations [20]. However, its performance can depend on large batch sizes, sufficient negative-sample diversity, and carefully designed augmentations.

Non-contrastive methods avoid the explicit use of negative samples. SimSiam employs a Siamese architecture with a prediction head and a stop-gradient operation to prevent representation collapse [21]. Barlow Twins instead computes the cross-correlation matrix between embeddings obtained from paired views and encourages it to approach the identity matrix [22]. This objective simultaneously promotes agreement between corresponding feature dimensions and reduces redundancy across different dimensions. Nevertheless, these methods mainly constrain global representations and do not explicitly characterize the dimensional complexity of individual feature neighborhoods.

Reconstruction-based methods derive supervisory signals by recovering missing or transformed image content. Masked autoencoders reconstruct randomly masked image patches and have demonstrated strong scalability in visual pre-training [23]. However, pixel-level reconstruction may emphasize low-level appearance information that is not necessarily aligned with subtle disease-related patterns in retinal OCT images.

Local Feature Reconstruction (LFR) learns self-supervised representations by reconstructing random projections of the input data [24]. The use of random projections provides a flexible supervisory objective and reduces dependence on modality-specific augmentations or masking strategies. Nevertheless, this objective does not explicitly characterize the neighborhood-level geometry or intrinsic dimensionality of the learned representations.

Predictive representation learning shifts the prediction target from image pixels to latent features. The Image-based Joint-Embedding Predictive Architecture (I-JEPA) predicts the representations of target image regions from visible contextual regions, enabling the model to learn semantic image structures without directly reconstructing pixel values [25]. This formulation reduces reliance on handcrafted view transformations but does not directly constrain the local dimensional organization of the feature space.

Autoregressive pre-training has also been extended to large-scale visual and multimodal representation learning. Fini et al. proposed AIMv2, which combines a vision encoder with a multimodal autoregressive decoder to predict image patches and text tokens [26]. The resulting visual encoder can be transferred to a range of downstream recognition tasks. However, AIMv2 relies on large-scale multimodal autoregressive learning rather than an explicit geometric objective for local feature organization.

Overall, existing self-supervised methods provide complementary objectives based on contrastive discrimination, non-contrastive consistency, redundancy reduction, reconstruction, latent prediction, and autoregressive generation. However, these objectives generally do not explicitly estimate or regulate the local intrinsic dimensionality of feature neighborhoods. This limitation may become important when neighboring samples exhibit similar global appearance but differ in subtle localized structures.

In addition to these general-purpose approaches, several self-supervised learning methods have been specifically designed for OCT image analysis. For instance, Jannat et al. proposed Multi-OCT-SelfNet, which employs a SwinV2 transformer backbone with masked autoencoder-based pre-training and multi-source data fusion to learn transferable OCT representations across multiple datasets [27]. Unlike our RetiLID framework, which focuses on local intrinsic dimensionality regularization through LID estimation and the asymptotic Fisher-Rao distance, Multi-OCT-SelfNet emphasizes cross-dataset generalization through large-scale pre-training and data fusion. These OCT-specific methods represent a complementary direction to our work, and a systematic comparison with them is a promising avenue for future investigation.

## 3. Materials and Methods

### 3.1. Method Overview

We propose RetiLID, an OCT-oriented self-supervised representation learning framework for retinal OCT image classification. The proposed framework is built upon the SimCLR self-supervised learning architecture [20], while introducing a local intrinsic dimensionality regularization module to preserve the local geometric structure of the learned feature space. By jointly optimizing the contrastive learning objective and the proposed regularization term, RetiLID aims to alleviate local dimensional collapse while preserving subtle pathological information in retinal OCT representations.

**Figure 1:**
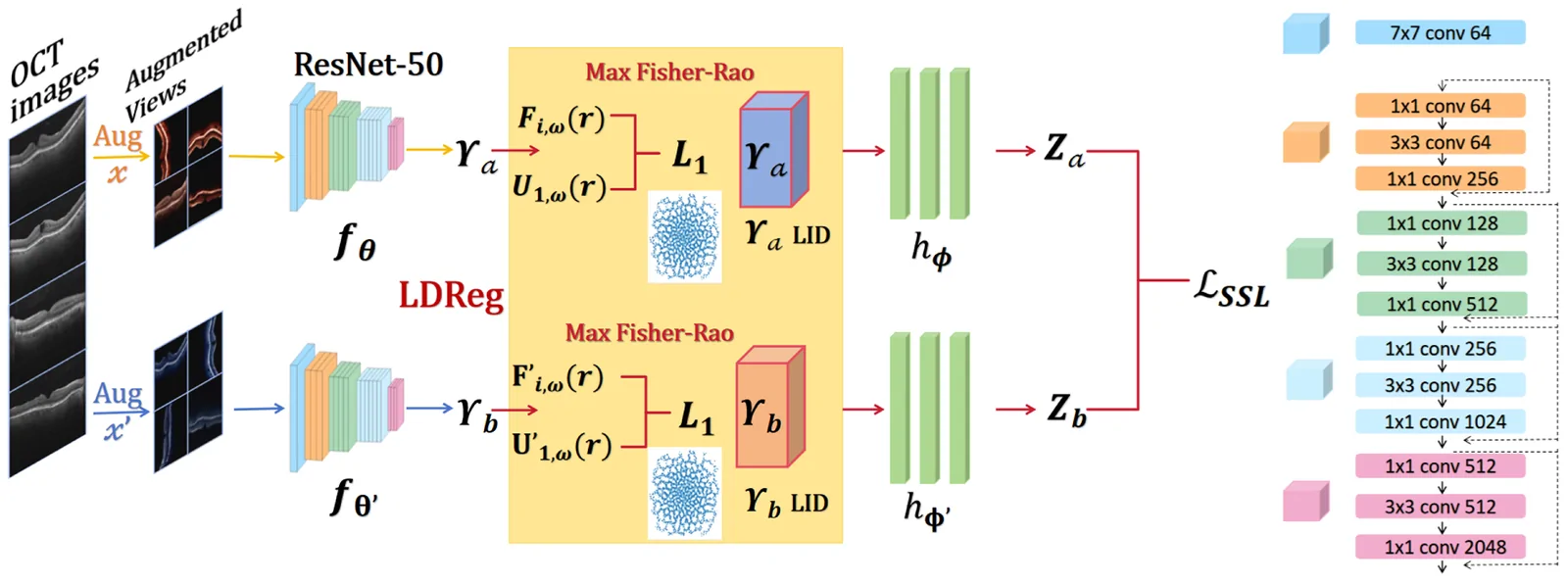
Self-supervised OCT image classification framework based on local intrinsic dimensionality regularization (RetiLID). The framework performs disease classification of unlabeled OCT images through image augmentation, channel-attention-enhanced ResNet-50 encoding, local dimensionality constraints combining the Fisher-Rao distance with $L_{1}$ regularization, and self-supervised loss optimization. The orange and blue arrows denote independent data augmentation and the input of the two correlated views into the ResNet-50 encoders. Red arrows indicate the computation and propagation related to the Fisher-Rao distance regularization, projection heads, and contrastive loss. Solid and dashed black arrows represent forward propagation and residual skip connections, respectively.

As illustrated in Figure 1, each unlabeled retinal OCT image is first transformed into two independently augmented views, denoted as *x* and $x^{\prime }$, using the data augmentation strategy described in Section 3.2. The two views are then simultaneously fed into a Siamese network consisting of two weight-sharing branches. Each branch comprises a ResNet-50 encoder $f_{\theta }$ [28] followed by a three-layer projection head $h_{\phi }$, where each fully connected layer contains 1024 hidden units. The encoder generates two feature representations, denoted as $Y_{a}$ and $Y_{b}$, while the projector maps them into embedding representations $Z_{a}$ and $Z_{b}$. The projected embeddings are optimized using the standard NT-Xent contrastive objective, denoted as $L_{\mathrm{SSL}}$, which serves as the backbone optimization objective during self-supervised pre-training.

To enhance the local discriminative capability of retinal OCT representations, the proposed RetiLID module is attached directly to the encoder outputs rather than the projection head. Specifically, the encoded representations $Y_{a}$ and $Y_{b}$ are simultaneously processed by a parallel local intrinsic dimensionality regularization branch. Let the neighborhood distance distributions induced by $Y_{a}$ and $Y_{b}$ be denoted as $F_{i}\left(r\right)$ and $F_{i}^{\prime }\left(r\right)$, respectively, with the corresponding uniform reference distributions denoted as $U_{i}\left(r\right)$ and $U_{i}^{\prime }\left(r\right)$. To alleviate local dimensional collapse, the framework maximizes the asymptotic Fisher-Rao distances between the local distance distributions and their corresponding uniform distributions, thereby encouraging higher local intrinsic dimensionality.

The local intrinsic dimensionality (LID) of each representation is estimated from its neighborhood distance distribution. Based on the estimated LID values, a local intrinsic dimensionality regularization term, denoted as $L_{1}$, is formulated using the asymptotic Fisher-Rao metric. To improve numerical stability, logarithmic scaling together with geometric-mean aggregation is further incorporated into the regularization formulation, reducing the influence of extreme LID values while preserving local geometric characteristics.

During optimization, the SimCLR contrastive loss $L_{\mathrm{SSL}}$ and the proposed local intrinsic dimensionality regularization term $L_{1}$ are jointly optimized to learn discriminative retinal OCT representations with well-preserved local geometry. The detailed formulations of the Fisher-Rao distance, LID estimation, the proposed regularization objective, and the overall optimization loss are presented in the following subsections.

### 3.2. Data Augmentation

To construct informative views for self-supervised learning, an OCT-specific stochastic augmentation strategy was applied to each retinal image. Two independently augmented views were generated using random horizontal flipping, rotation, brightness and contrast adjustment, and Gaussian noise injection. These transformations increase appearance diversity while minimizing distortion of the layered retinal anatomy and clinically relevant pathological patterns. The resulting view pairs are used for self-supervised representation learning and local intrinsic dimensionality regularization [9].

### 3.3. Fisher-Rao Distance-Based Regularization

To characterize the local geometry of the learned representation space, RetiLID employs the Fisher-Rao distance as the basis of its dimensionality regularization. The Fisher-Rao distance originates from information geometry and defines a Riemannian metric on a statistical manifold of probability distributions [29]. Its geodesic properties provide a principled means of measuring distributional differences in probability spaces [30]:

(1) $$d_{\mathrm{Fisher}\text{-}\mathrm{Rao}}\left(\theta_{1},\theta_{2}\right)=\underset{\gamma }{\inf}\int_{0}^{1}\sqrt{{\left(\frac{d\gamma (t)}{dt}\right)}^{\;T}I\left(\gamma (t)\right)\left(\frac{d\gamma (t)}{dt}\right)}dt,$$

where γ(t) is a smooth path connecting $\theta_{1}$ and $\theta_{2}$, with $\gamma \left(0\right)=\theta_{1}$ and $\gamma \left(1\right)=\theta_{2}$, and $I\left(\gamma (t)\right)$ denotes the Fisher information matrix evaluated along this path.

Although theoretically principled, direct computation of Equation (1) generally requires evaluating the Fisher information matrix and solving a geodesic optimization problem. This process is computationally expensive for high-dimensional feature representations. To address this issue, RetiLID characterizes the local distance distribution around each representation through its local intrinsic dimensionality. The resulting dimensional statistics enable the Fisher-Rao distance to be approximated in an asymptotic form, thereby avoiding explicit computation of the full Fisher information matrix and geodesic path.

The resulting approximation provides an efficient measure of local distributional differences. It is subsequently combined with the LID estimates introduced in the next subsection to formulate the local intrinsic dimensionality regularization objective.

### 3.4. Local Intrinsic Dimensionality (LID) Estimation

To characterize the local dimensional properties required by the Fisher-Rao distance-based regularization described above, we employ LID to measure the neighborhood complexity of the learned feature representations. Unlike global dimensionality measures, which primarily describe the overall structure of the representation space, LID focuses on the distribution surrounding each individual feature. It is therefore more sensitive to local dimensional degeneration and redundant feature structures [31,32]. Such local characterization is particularly relevant to retinal OCT images, where diagnostically important information is often associated with subtle variations in retinal layers and local pathological structures.

For an arbitrary point x in the feature representation space, the set of samples within its *K*-nearest local neighborhood is denoted as $N_{k}\left(x\right)=\left\{x_{1},x_{2},\ldots ,x_{k}\right\}$. For each neighboring feature $x_{i}$, the corresponding neighborhood distance is defined as $r_{i}=d\left(x,x_{i}\right)$, *i* = 1, 2, …, *k*, where d(·,·) denotes the distance metric between individual feature representations. After arranging the distances in ascending order, they satisfy $0<r_{1}\leq r_{2}\leq \cdots \leq r_{k}$. Accordingly, $r_{i}$ represents the distance from x to its *i*-th nearest neighbor, while $r_{k}$ is the distance to the farthest neighbor within the selected *k*-nearest-neighbor set and therefore defines the radius of the local neighborhood. Then the maximum likelihood estimator of LID is defined as:

(2) $$\hat{LID}\left(x\right)={\left(\frac{1}{k-1}\sum_{i=1}^{k-1}\ln\frac{r_{k}}{r_{i}}\right)}^{-1}.$$

Here, $\hat{\mathrm{LID}}\left(x\right)$ is a scalar estimate of the local intrinsic dimensionality around x. The hat symbol indicates that the true local dimensionality is unknown and is estimated from a finite set of neighboring representations. In Equation (2), each of the first k−1 distances is normalized by $r_{k}$, making the estimator dependent on relative neighborhood expansion rather than the absolute scale of the feature space. As a result, uniformly scaling all neighborhood distances does not change the estimated LID.

The estimator is theoretically motivated by the local power-law behavior of neighborhood distances. Let *R* denote the random distance from x to a neighboring representation. Within a sufficiently small neighborhood, the cumulative distance distribution can be approximated as $P\left(R\leq r\right)\propto r^{\mathrm{LID}}$. Under this assumption, the exponent determines the rate at which the local probability mass increases with the neighborhood radius. The logarithmic averaging formulation in Equation (2) provides an asymptotic estimate of this exponent from the observed neighborhood distances.

A lower value of $\hat{\mathrm{LID}}\left(x\right)$ indicates that the neighboring representations are concentrated within a relatively low-dimensional local structure. By contrast, a higher value suggests that the local neighborhood contains variations along a larger number of effective directions. LID is not restricted to integer values because it describes the expansion behavior of a local probability distribution rather than the dimensionality of an ideal linear subspace. In the context of representation learning, extremely low LID values may indicate that locally distinct samples have been mapped to overly similar features, whereas sufficiently high LID values generally correspond to a richer local representation structure.

During training, LID is estimated from the encoder outputs $Y_{a}$ and $Y_{b}$. Each representation is treated as a query point, and its neighboring features are selected from the representations available in the current feature set. The resulting sample-wise LID values provide a local geometric description complementary to the self-supervised objective computed from the projected embeddings $Z_{a}$ and $Z_{b}$.

In addition to the finite-neighborhood estimator, two neighborhood statistics are calculated to obtain the limiting LID required by the asymptotic Fisher-Rao formulation. Let $\mu_{k}$ denote the mean distance from the current representation to its *k* nearest neighbors, and let $\omega_{k}$ denote the radius of the selected local neighborhood:

(3) $$\mu_{k}=\frac{1}{k}\sum_{i=1}^{k}r_{i},\;\omega_{k}=r_{k}.$$

The quantity $\mu_{k}$ summarizes the average spatial extent of the neighboring representations, whereas $\omega_{k}$ specifies the outer boundary of the local neighborhood. Their relative difference reflects how the neighborhood distances are distributed between the query representation and the neighborhood boundary.

Let $F_{\omega }\left(r\right)$ denote the local distance distribution restricted to the measurement interval [0,ω]. The limiting LID associated with this distribution is defined as

(4) $${\mathrm{LID}}_{F_{\omega }}^{*}≜\lim_{r\to 0^{+}}{\mathrm{LID}}_{F_{\omega }}\left(r\right)=-\frac{\mu_{k}}{\mu_{k}-\omega_{k}}=\frac{\mu_{k}}{\omega_{k}-\mu_{k}}.$$

Since $\mu_{k}<\omega_{k}$ for a non-degenerate neighborhood, ${\mathrm{LID}}_{F_{\omega }}^{*}$ is positive. This quantity characterizes the limiting dimensional behavior of the local distance distribution and serves as the sample-wise dimensional statistic used in the subsequent asymptotic Fisher-Rao regularization.

It is important to distinguish the roles of Equations (2) and (4). Equation (2) provides an empirical LID estimate from a finite set of *k* neighboring representations, whereas Equation (4) describes the limiting LID associated with the local distance distribution. The latter is used to construct the regularization terms and the overall optimization objective presented in the following subsection.

### 3.5. Loss Function

Using the limiting LID defined in Equation (4), the regularization branch evaluates the local distance distributions associated with the two encoder representations $Y_{a}$ and $Y_{b}$. For the *i*-th image pair in a training batch, let $F_{i,\omega }\left(r\right)$ and $F_{i,\omega }^{\prime }\left(r\right)$ denote the local distance distributions induced by $Y_{a,i}$ and $Y_{b,i}$, respectively, over the measurement interval [0,ω]. Their corresponding one-dimensional uniform reference distributions are denoted by $U_{1,\omega }\left(r\right)$ and $U_{1,\omega }^{\prime }\left(r\right)$.

The sample-wise asymptotic Fisher-Rao distances for the two representation branches are defined as

(5) $$\begin{array}{cc} \Delta_{i}^{a} & =\lim_{\omega \to 0}d_{\mathrm{AFR}}\left(F_{i,\omega }\left(r\right),U_{1,\omega }\left(r\right)\right)=\left|\ln{\mathrm{LID}}_{F_{i,\omega }}^{*}\right|,\\ \Delta_{i}^{b} & =\lim_{\omega \to 0}d_{\mathrm{AFR}}\left(F_{i,\omega }^{\prime }\left(r\right),U_{1,\omega }^{\prime }\left(r\right)\right)=\left|\ln{\mathrm{LID}}_{F_{i,\omega }^{\prime }}^{*}\right|, \end{array}$$

where $d_{\mathrm{AFR}}\left(\cdot ,\cdot \right)$ denotes the asymptotic Fisher-Rao distance. The quantities ${\mathrm{LID}}_{F_{i,\omega }}^{*}$ and ${\mathrm{LID}}_{F_{i,\omega }^{\prime }}^{*}$ are the limiting LID values of the local distance distributions associated with $Y_{a,i}$ and $Y_{b,i}$, respectively. Accordingly, $\Delta_{i}^{a}$ and $\Delta_{i}^{b}$ are nonnegative scalars that measure the geometric discrepancies between the two local distance distributions and their corresponding one-dimensional uniform reference distributions.

To jointly regularize the local geometry of both representation branches, the proposed $L_{1}$ regularization term aggregates the squared magnitudes of the sample-wise asymptotic Fisher-Rao distances:

(6) $$L_{L_{1}}={\left[\frac{1}{2N}\sum_{i=1}^{N}\left({\left(\Delta_{i}^{a}\right)}^{2}+{\left(\Delta_{i}^{b}\right)}^{2}\right)\right]}^{\frac{1}{2}},$$

which can be equivalently expressed as

(7) $$L_{L_{1}}={\left[\frac{1}{2N}\sum_{i=1}^{N}\left({\left(\ln{\mathrm{LID}}_{F_{i,\omega }}^{*}\right)}^{2}+{\left(\ln{\mathrm{LID}}_{F_{i,\omega }^{\prime }}^{*}\right)}^{2}\right)\right]}^{\frac{1}{2}}.$$

Here, *N* denotes the number of paired representations in the training batch. The squared terms assign greater importance to representations exhibiting larger local distributional discrepancies, while the outer square root preserves the scale of the aggregated distances. Consequently, $L_{L_{1}}$ is a batch-level scalar that summarizes the local dimensional characteristics of both encoder branches. In this study, $L_{1}$ denotes the name of the proposed regularization strategy rather than conventional $L_{1}$-norm regularization.

In parallel with the regularization branch, we adopt the standard SimCLR contrastive learning objective. Given a batch of unlabeled retinal OCT images, each image is independently augmented to generate two correlated views, denoted as *x* and $x^{\prime }$. The two augmented views are then simultaneously fed into a Siamese network with shared encoder parameters $f_{\theta }$, yielding the corresponding feature representations:

(8) $$Y_{a}=f_{\theta }\left(x\right),\;Y_{b}=f_{\theta }\left(x^{\prime }\right).$$

The encoded representations are subsequently mapped to the embedding space through the shared-weight projector $h_{\phi }$, yielding

(9) $$Z_{a}=h_{\phi }\left(Y_{a}\right),\;Z_{b}=h_{\phi }\left(Y_{b}\right).$$

The projected embeddings $Z_{a}$ and $Z_{b}$ are used to compute the base SimCLR contrastive loss $L_{\mathrm{SSL}}$, which is the standard NT-Xent objective:

(10) $$L_{\mathrm{SSL}}=-\frac{1}{2N}\sum_{i=1}^{N}\left[\log\frac{\exp(\mathrm{sim}\left(Z_{a}^{i},Z_{b}^{i}\right)/\tau )}{\sum_{k\neq i}\exp\left(\mathrm{sim}\left(Z_{a}^{i},Z_{k}\right)/\tau \right)}+\log\frac{\exp(\mathrm{sim}\left(Z_{b}^{i},Z_{a}^{i}\right)/\tau )}{\sum_{k\neq i}\exp\left(\mathrm{sim}\left(Z_{b}^{i},Z_{k}\right)/\tau \right)}\right],$$

where $\mathrm{sim}\left(u,v\right)=u^{⊤}v/\left(∥u∥∥v∥\right)$ is the cosine similarity, τ is a temperature parameter, and $Z_{k}$ denotes all embeddings in the batch except the anchor itself.

The overall optimization objective of RetiLID is formulated as

(11) $$L_{\mathrm{RetiLID}}=L_{\mathrm{SSL}}-\beta L_{L_{1}},$$

or, equivalently,

(12) $$L_{\mathrm{RetiLID}}=L_{\mathrm{SSL}}-\beta {\left[\frac{1}{2N}\sum_{i=1}^{N}\left({\left(\ln{\mathrm{LID}}_{F_{i,\omega }}^{*}\right)}^{2}+{\left(\ln{\mathrm{LID}}_{F_{i,\omega }^{\prime }}^{*}\right)}^{2}\right)\right]}^{\frac{1}{2}},$$

where β≥0 is a weighting coefficient that controls the contribution of the local intrinsic dimensionality regularization relative to the self-supervised learning objective.

Since $L_{\mathrm{RetiLID}}$ is minimized during training, the negative sign preceding $L_{L_{1}}$ encourages larger asymptotic Fisher-Rao distances between the learned local distance distributions and their one-dimensional uniform references. Therefore, the two representation branches are simultaneously encouraged to preserve richer local dimensional structures, while $L_{\mathrm{SSL}}$ maintains the consistency and discriminative properties of the paired embeddings.

## 4. Results

### 4.1. Dataset

To evaluate the proposed RetiLID framework, experiments were conducted on three public retinal OCT datasets: TMI, BOE, and CELL.

- TMI dataset: This dataset was collected from Noor Eye Hospital in Tehran [33]. It consists of 3799 OCT images from three categories: AMD, DME, and NORMAL. Specifically, the dataset contains 1362 AMD images, 852 DME images, and 1585 NORMAL images. Similar to BOE, TMI is also used for three-class retinal disease classification.
- BOE dataset: This dataset contains 3231 OCT images collected from 45 subjects by Srinivasan et al. [2]. It includes three categories, namely AMD, DME, and NORMAL, with 723, 1101, and 1407 images, respectively. Therefore, BOE is used for a three-class OCT classification task.
- CELL dataset: This dataset is a large-scale multi-institutional retinal OCT dataset collected from 5319 adult patients [3]. It contains 109,309 OCT images from four categories, including CNV, DME, DRUSEN, and NORMAL, with 37,455, 11,598, 8866, and 51,390 images, respectively. Thus, CELL is used for a four-class classification task.

**Figure 2:**
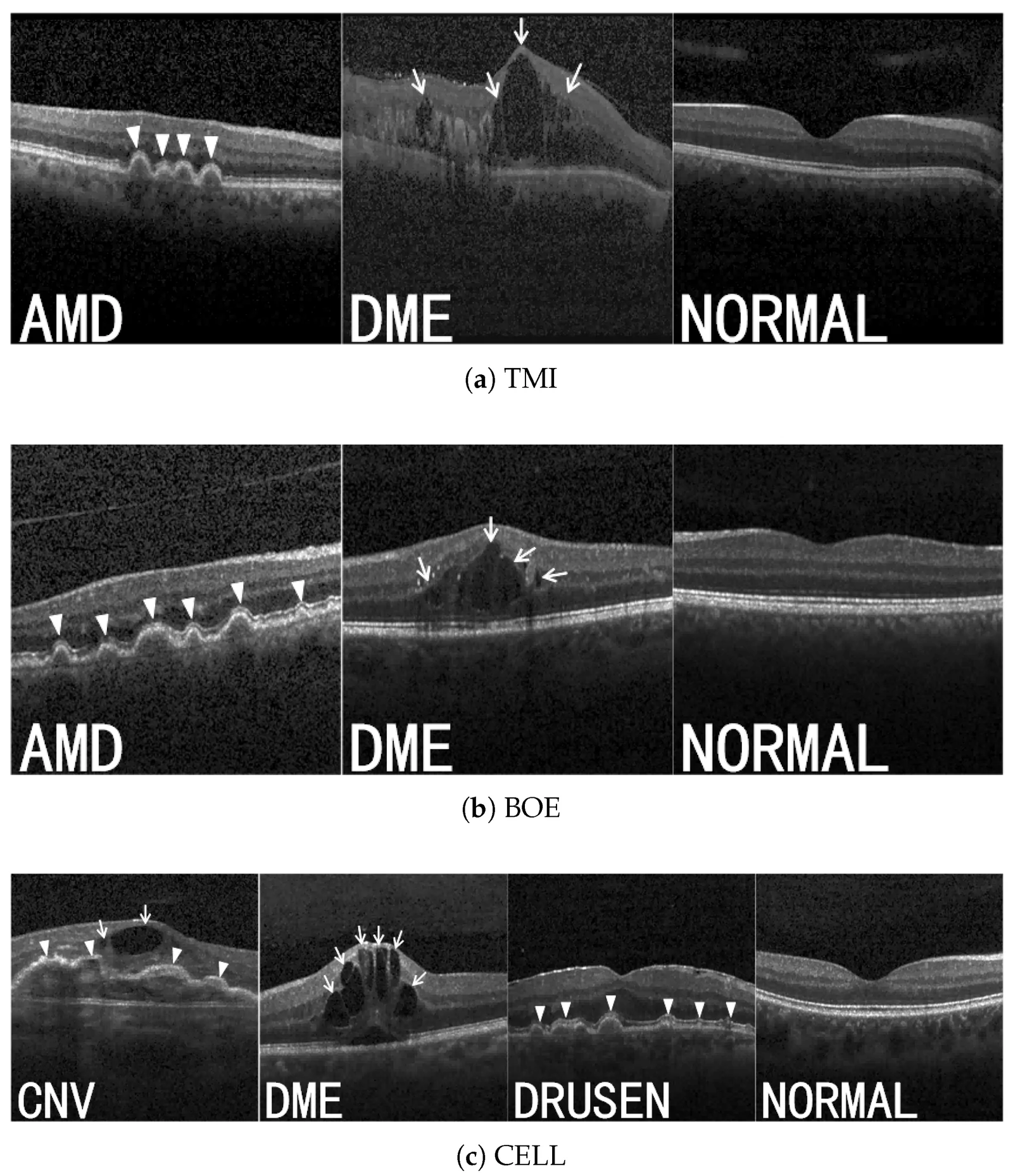
Examples of retinal lesions from the three OCT datasets used in this study. White arrows indicate retinal edema and intraretinal cystic changes in DME, while white triangles indicate characteristic abnormal retinal structural changes in AMD, CNV, and DRUSEN images.

For consistency across datasets and to meet the input requirements of the ResNet-based backbone, all OCT images were resized to 224×224×3 after data augmentation. Representative images from the three datasets are shown in Figure 2, illustrating different retinal conditions.

### 4.2. Implementation Details

The experiments consist of two successive stages: self-supervised pre-training and linear evaluation. Unless otherwise specified, a consistent training protocol is adopted across all datasets.

#### 4.2.1. Dataset Splits and Data Usage

All three datasets adopt their officially released data splits without any custom re-partitioning. The BOE dataset contains 3231 OCT B-scans from 45 subjects and follows the official train/validation split (2248/983). The TMI dataset contains 3799 OCT B-scans and adopts the official train/validation/test split (2382/1026/390). The CELL dataset contains 109,309 OCT B-scans from 5319 patients and follows the official train/validation split (108,242/1067). The BOE and TMI datasets are divided at the patient level, ensuring that images from the same patient do not appear in both the training and validation/test sets. The CELL dataset follows the official image-level split released by Kermany et al. The detailed dataset statistics and split configurations are summarized in Table 1.

It is worth noting that for the BOE and CELL datasets, the official validation splits serve as the test sets for final performance evaluation, as no separate test partitions are provided in the official releases; the splits are named as “validation” in the original benchmarks. For the TMI dataset, the official released split contains 2382 images for training, 1026 for validation, and 390 for testing. The sum of the three splits is 3798, although the original publication reports 3799 images in total. This minor discrepancy originates from the publicly released dataset rather than our experimental protocol. In our experiments, we adhere strictly to the actual composition of the released dataset and conduct all experiments based on the 3798 images provided in the official split.

#### 4.2.2. Self-Supervised Pre-Training

During self-supervised pre-training, the ResNet-50 backbone is trained with a batch size of 16. Each unlabeled OCT image is processed using the augmentation strategy described in Section 3.2 and converted to a unified input size of 224×224×3. The model is optimized using stochastic gradient descent (SGD) with a momentum of 0.9 and a weight decay of $1\times 10^{-4}$. The initial learning rate is set to 0.05×batch_size/256 and adjusted using a cosine annealing schedule without warm-up. The pre-training is performed for 150 epochs. Following the standard SimCLR training strategy, each retinal OCT image is independently augmented using RandomResizedCrop (224, scale = 0.2–1.0), RandomHorizontalFlip, ColorJitter (0.4, 0.4, 0.4, 0.2), RandomGrayscale (p=0.2), and GaussianBlur (σ=0.1–2.0, p=0.5). The complete pre-training hyperparameters are summarized in Table 2.

The neighborhood size *k*, regularization weight β, and relative learning rate are configured separately for each dataset: k=32, β=0.005, learning rate =1×baselr for TMI; k=128, β=0.01, learning rate =0.5×baselr for BOE; and k=32, β=0.005, learning rate =2×baselr for CELL.

#### 4.2.3. Linear Evaluation

During linear evaluation, the pre-trained encoder is frozen, and only a linear classification layer is optimized using stochastic gradient descent. The initial learning rate is set to 0.3, the batch size is 1024, and the classifier is trained for 100 epochs using a cosine annealing schedule. For training, the input images are processed using random resized cropping to 224×224 and random horizontal flipping. For validation, each image is first resized to 256×256 and then center-cropped to 224×224. This deterministic validation pipeline ensures consistent input dimensions without introducing stochastic transformations during evaluation. The complete linear evaluation settings are summarized in Table 3.

For the semi-supervised setting, labeled samples are randomly selected from the training set using fixed random seeds. Approximately 22, 24, and 1082 labeled images are used for the 1% setting on BOE, TMI, and CELL, respectively, while approximately 224, 238, and 10,824 labeled images are used for the corresponding 10% setting. All results are reported as mean ± standard deviation over multiple independent runs, as detailed in Table 1.

#### 4.2.4. Hardware Platform and Computational Cost

All experiments were performed on the high-performance computing platform of the Institute of Smart Healthcare, Nanjing University of Information Science and Technology. The platform is equipped with NVIDIA A100, RTX 4090, and RTX 3090 GPUs and provides more than 2 PB of storage. Under the experimental configuration described in this study, the self-supervised pre-training required approximately 1.2 h for the TMI dataset, 1.0 h for the BOE dataset, and 18.5 h for the CELL dataset. Linear evaluation required approximately 6–10 min per experiment, while semi-supervised fine-tuning required approximately 10–15 min. The average inference time for a single OCT image was approximately 8 ms on an NVIDIA RTX 4090 GPU.

### 4.3. Experimental Results

In this section, we evaluate the proposed RetiLID framework against representative self-supervised learning methods, including SimCLR [20], SimSiam [21], BarlowTwins [22], LFR [24], AimV2 [26], and I-JEPA [25]. To ensure a fair comparison, all competing methods are evaluated using the same experimental protocol and linear evaluation setting. Performance is assessed using classification accuracy, sensitivity, and specificity. Receiver operating characteristic (ROC) and precision–recall (PR) curves are also presented to examine the discriminative performance of the learned representations across different decision thresholds.

The quantitative results on the three datasets are reported in Table 4, Table 5 and Table 6. Overall, RetiLID achieves competitive classification performance among the compared self-supervised methods, obtaining accuracies of 94.35%, 92.48% and 92.56% on the TMI, BOE, and CELL datasets, respectively. The consistent performance across the three datasets indicates that local intrinsic dimensionality regularization improves the discriminative quality of self-supervised OCT representations.

**Figure 3:**
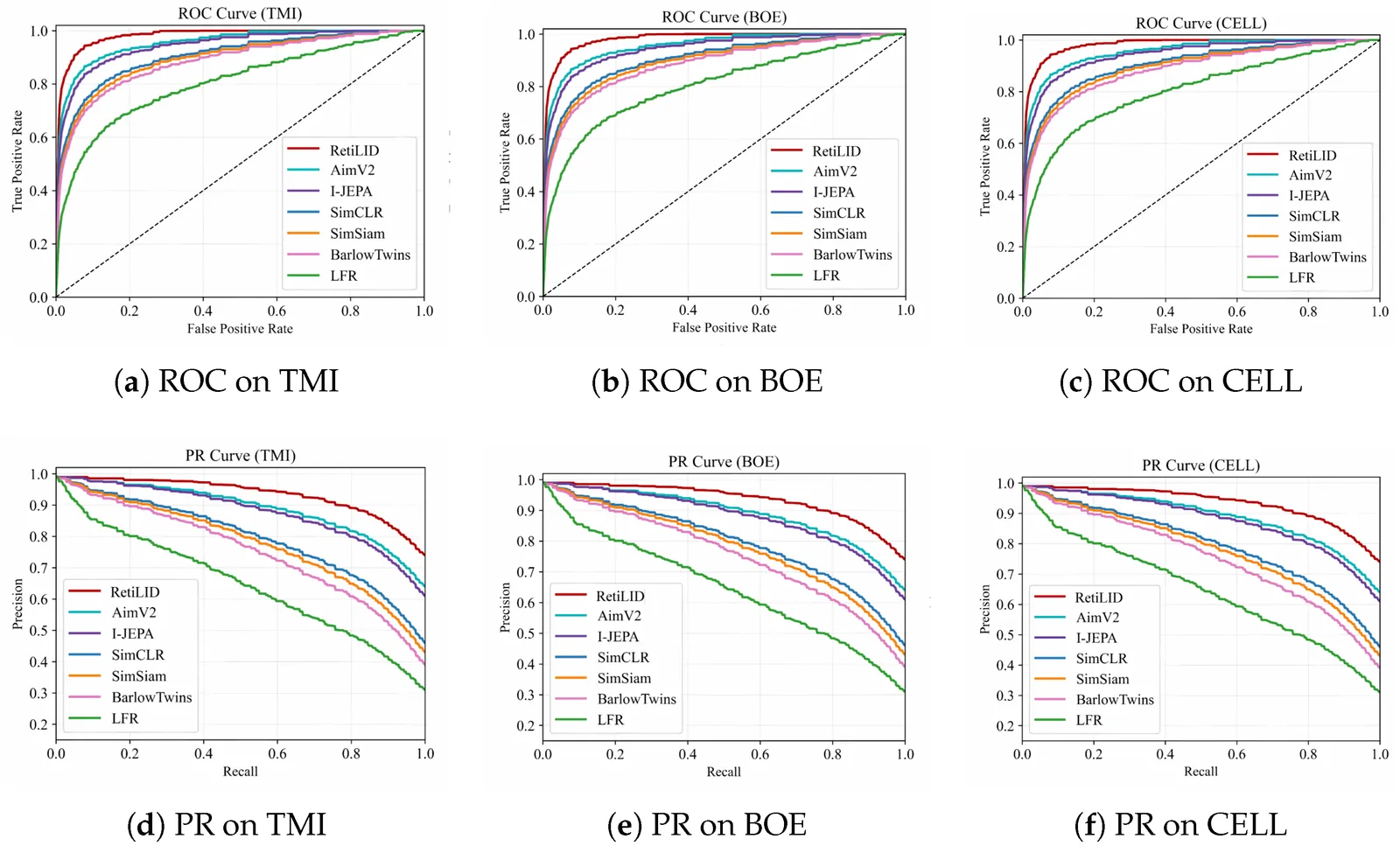
Receiver operating characteristic (ROC) and precision–recall (PR) curves obtained by linear evaluation of the pre-trained models on the TMI, BOE, and CELL datasets.

**Figure 4:**
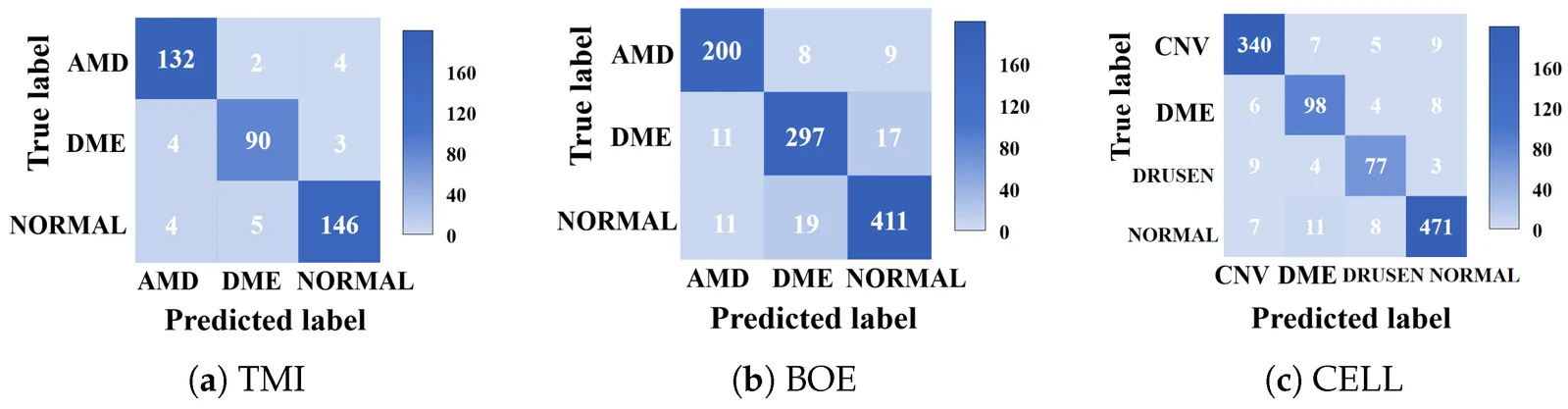
Confusion matrices obtained by linear evaluation on the TMI, BOE, and CELL datasets.

Figure 3 illustrates the ROC and PR curves obtained through linear evaluation of the pre-trained representations. Compared with the competing approaches, RetiLID generally produces more favorable curves, demonstrating stronger discrimination across a range of classification thresholds. Figure 4 further presents the confusion matrices of RetiLID, providing a class-wise analysis of its prediction performance and revealing the remaining misclassification patterns among different retinal disease categories.

### 4.4. Hyperparameter Sensitivity Analysis

We investigate the sensitivity of RetiLID to two key hyperparameters, namely the neighborhood size *k* and the regularization weight β. In the proposed framework, *k* determines the number of nearest neighbors used to characterize the local feature distribution and estimate its intrinsic dimensionality, whereas β controls the contribution of the local intrinsic dimensionality regularization relative to the self-supervised learning objective.

The sensitivity analysis is conducted using the candidate sets k∈{16,32,64,128} and $\beta \in \{0.1,0.05,0.01,0.005,0.001,5\times 10^{-6}\}$. Following a one-factor-at-a-time strategy, one hyperparameter is varied while the other is fixed at its selected value. This procedure allows the individual influence of *k* and β on downstream classification performance to be examined separately.

The results show that model performance is sensitive to both hyperparameters. An excessively small *k* may provide an insufficient number of neighboring representations for reliable local dimensionality estimation, whereas an overly large *k* may incorporate samples outside the relevant local structure. Similarly, a small β may provide insufficient regularization, while an excessively large value may cause the local intrinsic dimensionality term to dominate the self-supervised learning objective. These observations indicate that an appropriate balance between neighborhood scale and regularization strength is important for learning discriminative OCT representations.

Misclassification between DRUSEN and CNV. It is also worth noting that a certain number of DRUSEN samples in the CELL dataset were misclassified as CNV, as shown in Figure 4c. We attribute this phenomenon to several factors. From the perspective of OCT imaging, both DRUSEN and CNV typically appear as hyper-reflective lesions beneath the retinal pigment epithelium (RPE), and their morphological characteristics may partially overlap, especially in early or atypical stages, which poses a natural challenge for automated classifiers. In addition, the CELL dataset contains a substantially smaller number of DRUSEN samples (8866 images) compared to CNV (37,455 images), which may introduce class imbalance and bias the model toward the majority category when visual features are similar. Furthermore, some misclassified DRUSEN samples exhibit ambiguous morphological patterns that lie near the decision boundary between DRUSEN and CNV, making accurate discrimination inherently difficult. These observations suggest that incorporating a more balanced training set and exploring more fine-grained lesion-specific features could further reduce such misclassifications in future work.

**Figure 5:**
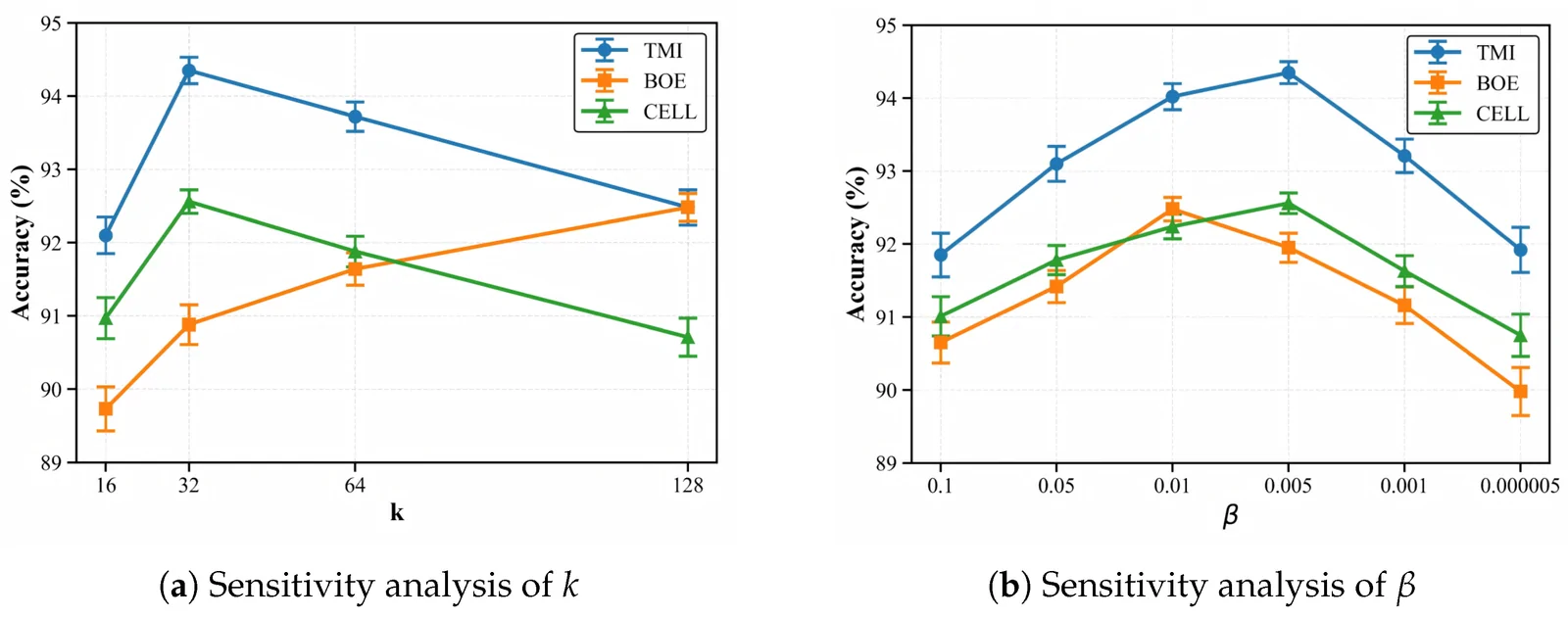
Hyperparameter sensitivity analysis of *k* and β on the TMI, BOE, and CELL datasets.

The selected hyperparameter settings are k=32 and β=0.005 for TMI, k=128 and β=0.01 for BOE, and k=32 and β=0.005 for CELL. The corresponding sensitivity results are presented in Figure 5 and Table 7 and Table 8.

### 4.5. Ablation Study

To systematically evaluate the contribution of each component of the proposed RetiLID framework, we conducted a component-wise ablation study on the TMI dataset by progressively introducing each key component into the baseline SimCLR framework. Four experimental configurations were considered: (i) the baseline SimCLR model without local intrinsic dimensionality regularization, (ii) the baseline model with direct Local Intrinsic Dimensionality (LID) maximization, (iii) the complete framework using the proposed asymptotic Fisher-Rao regularization with the $L_{1}$ formulation, and (iv) the corresponding $L_{2}$ formulation. Here, $L_{1}$ denotes the absolute summation formulation of the asymptotic Fisher-Rao regularization, while $L_{2}$ denotes the squared-aggregation-then-square-root formulation. All configurations were evaluated under the same linear evaluation protocol with 100%, 10%, and 1% labeled data. The experimental results on the TMI dataset are summarized in Table 9.

As shown in Table 9, incorporating LID estimation yields a modest improvement over the baseline SimCLR. After incorporating the proposed asymptotic Fisher-Rao regularization, further performance gains are observed. Specifically, the Fisher-Rao ($L_{1}$) formulation achieves balanced performance across different supervision levels, yielding improvements of +1.49%, +1.07%, and +1.56% over the baseline under the 100%, 10%, and 1% labeled settings, respectively. Comparatively, the $L_{2}$ formulation yields slightly lower or comparable accuracy under these settings. Overall, the ablation study on the TMI dataset demonstrates that both LID estimation and the asymptotic Fisher-Rao regularization contribute positively to the proposed RetiLID framework. The logarithmic Fisher-Rao formulation further enhances the effectiveness of local intrinsic dimensionality regularization, particularly when only limited labeled data are available.

### 4.6. Comparison with Original LDReg

To further distinguish our proposed RetiLID framework from the original LDReg method [13], we additionally implemented the original LDReg framework using its original SimSiam-based self-supervised setting [13]. We evaluated this baseline on the same retinal OCT datasets under the identical linear evaluation protocol adopted in this work. As shown in Table 10, although the original LDReg already improves representation learning compared with the vanilla SimSiam baseline, our OCT-oriented RetiLID consistently achieves better performance across all three datasets. These results provide empirical evidence that our performance improvement does not simply originate from applying the local dimensionality regularization theory itself, but from the proposed OCT-oriented adaptation, including the reformulated optimization objective, retinal-specific training pipeline, and the integration of the regularization into OCT representation learning.

### 4.7. Analysis of Learned LID Distributions

To investigate the geometric effect of the proposed local dimensionality regularization, we analyzed the distributions of the learned Local Intrinsic Dimensionality (LID) values on the BOE dataset. Specifically, we compared the baseline SimCLR model, SimCLR with direct LID maximization, and the complete framework with the proposed asymptotic Fisher-Rao regularization.

**Figure 6:**
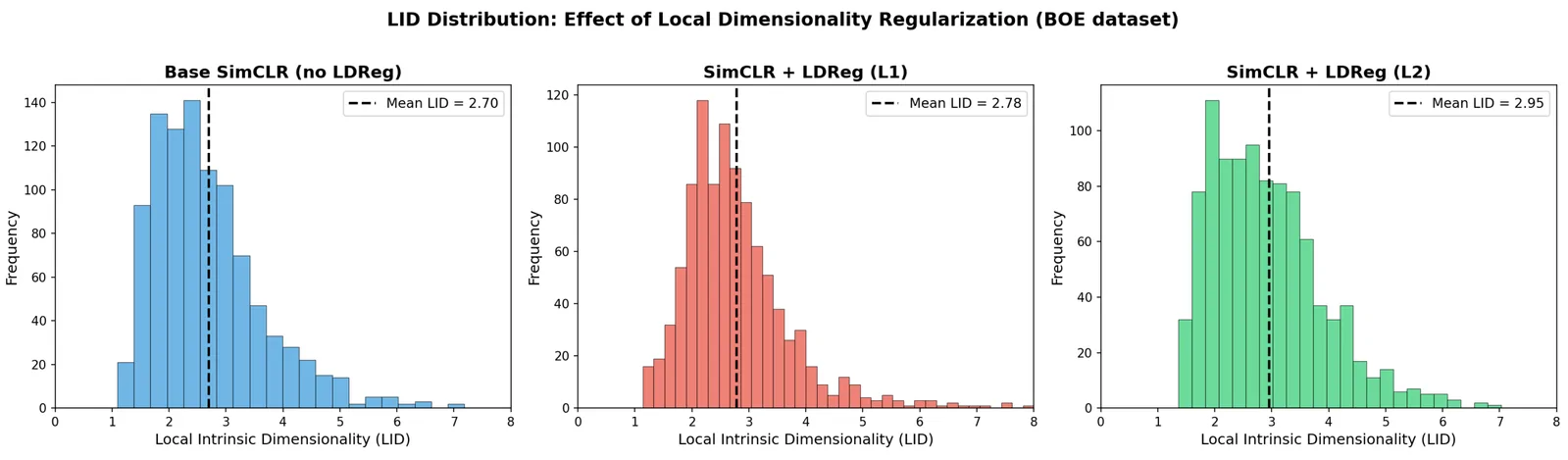
Effect of local dimensionality regularization on the learned Local Intrinsic Dimensionality (LID) distributions of retinal OCT representations on the BOE dataset.

As shown in Figure 6, the distribution of learned LID values progressively shifts toward larger intrinsic dimensionalities after introducing local dimensionality regularization. The mean LID increases from 2.70 for the baseline SimCLR model to 2.78 after directly maximizing the estimated LID, and further to 2.95 after incorporating the proposed asymptotic Fisher-Rao regularization. This progressive increase indicates that the proposed regularization effectively enlarges the local intrinsic dimensionality of the learned feature space, thereby alleviating local dimensional collapse.

Moreover, the observed changes in the LID distributions are qualitatively consistent with the improvements in downstream classification performance. As the average LID increases, the model benefits from richer local geometric structures, leading to more discriminative retinal OCT representations. Compared with direct LID maximization, the Fisher-Rao regularization produces a larger increase in the learned intrinsic dimensionality while simultaneously yielding superior classification performance, providing further evidence that the proposed regularization improves feature geometry rather than merely optimizing the classification objective.

These LID distribution analyses provide direct experimental support for the proposed mechanism and demonstrate that the effectiveness of the framework originates from its ability to preserve the local geometric structure of retinal OCT representations through local intrinsic dimensionality regularization.

### 4.8. Interpretability Analysis via Grad-CAM

**Figure 7:**
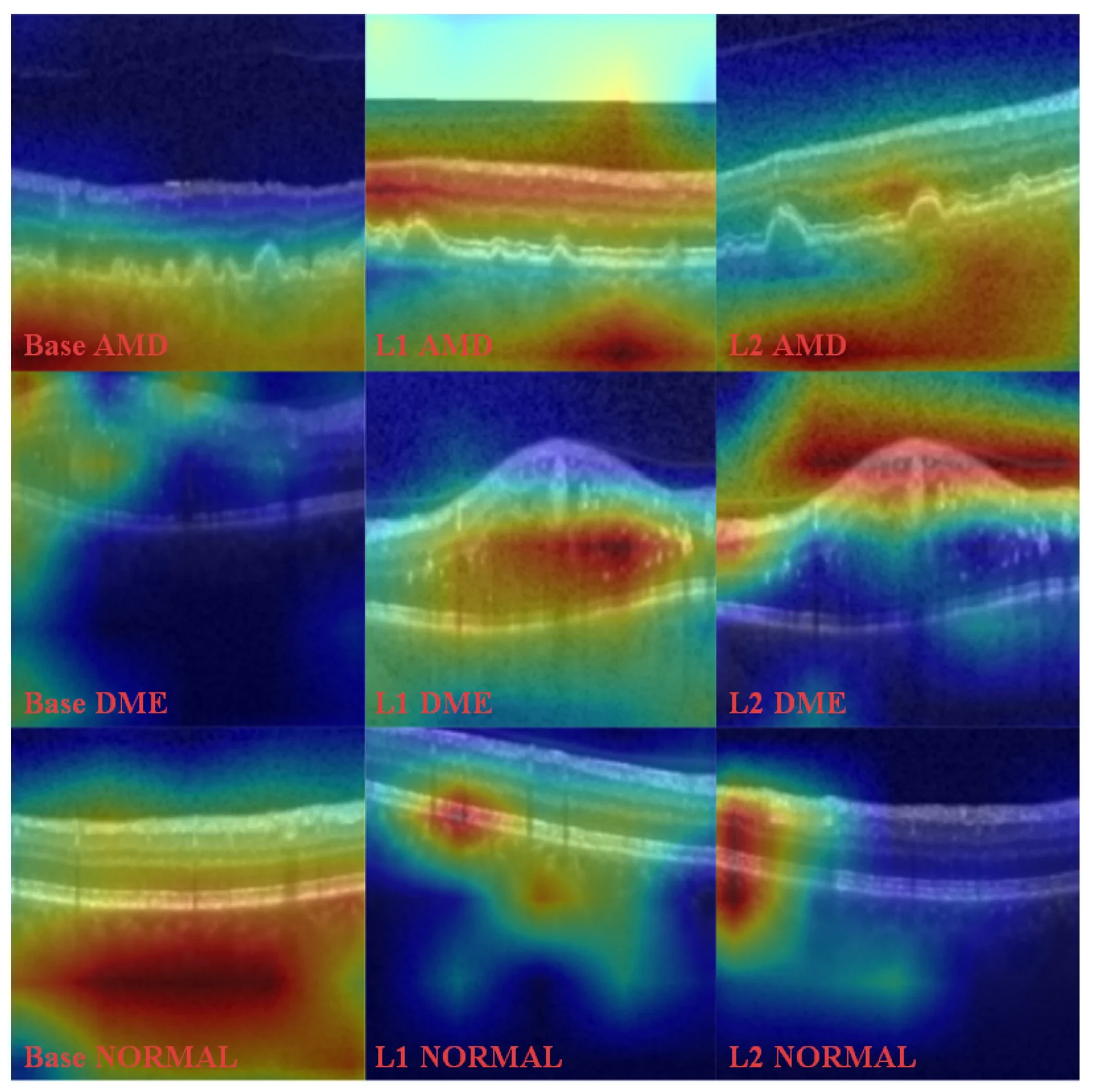
Grad-CAM visualizations of different model configurations on representative TMI samples across AMD, DME, and NORMAL categories. The heatmaps highlight regions most influential to the model’s classification decisions. The colors in the Grad-CAM heatmaps indicate the relative importance of different image regions to the model’s prediction. The importance progressively decreases from red (highest importance) to yellow, yellow-green/green, blue, and finally dark blue (lowest importance).

To further investigate the interpretability of the proposed framework and provide visual evidence of its decision-making behavior, we conducted a Grad-CAM visualization study on representative OCT samples from the TMI dataset. We compared three model configurations: (i) the baseline SimCLR model; (ii) the proposed RetiLID with the $L_{1}$ regularization formulation, which aggregates sample-wise distances using the absolute summation of logarithmic LID values; and (iii) RetiLID with the $L_{2}$ formulation, which adopts the squared-aggregation-then-square-root strategy. The corresponding Grad-CAM visualizations are presented in Figure 7.

As shown in Figure 7, the baseline SimCLR model exhibits a notable Clever Hans effect on AMD samples, where the attention map is predominantly concentrated in the vitreous region, largely bypassing the retinal pigment epithelium (RPE) layer in which pathological changes typically manifest. This indicates that the baseline model may rely on spurious correlations or image artifacts for classification. In contrast, for DME and NORMAL samples, all three models exhibit reasonable attention to the sub-retinal cystic regions and retinal layer structures, respectively, showing that the models are capable of capturing clinically plausible anatomical and pathological patterns.

Comparing the two regularized variants, RetiLID ($L_{1}$) produces more structurally aligned attention maps, with heatmaps concentrated along the retinal layers for both DME and NORMAL samples. RetiLID ($L_{2}$), while exhibiting improved attention over the baseline SimCLR, still presents a notable upward shift in AMD samples, suggesting that the $L_{1}$ formulation provides more consistent and interpretable localization performance.

**Figure 8:**
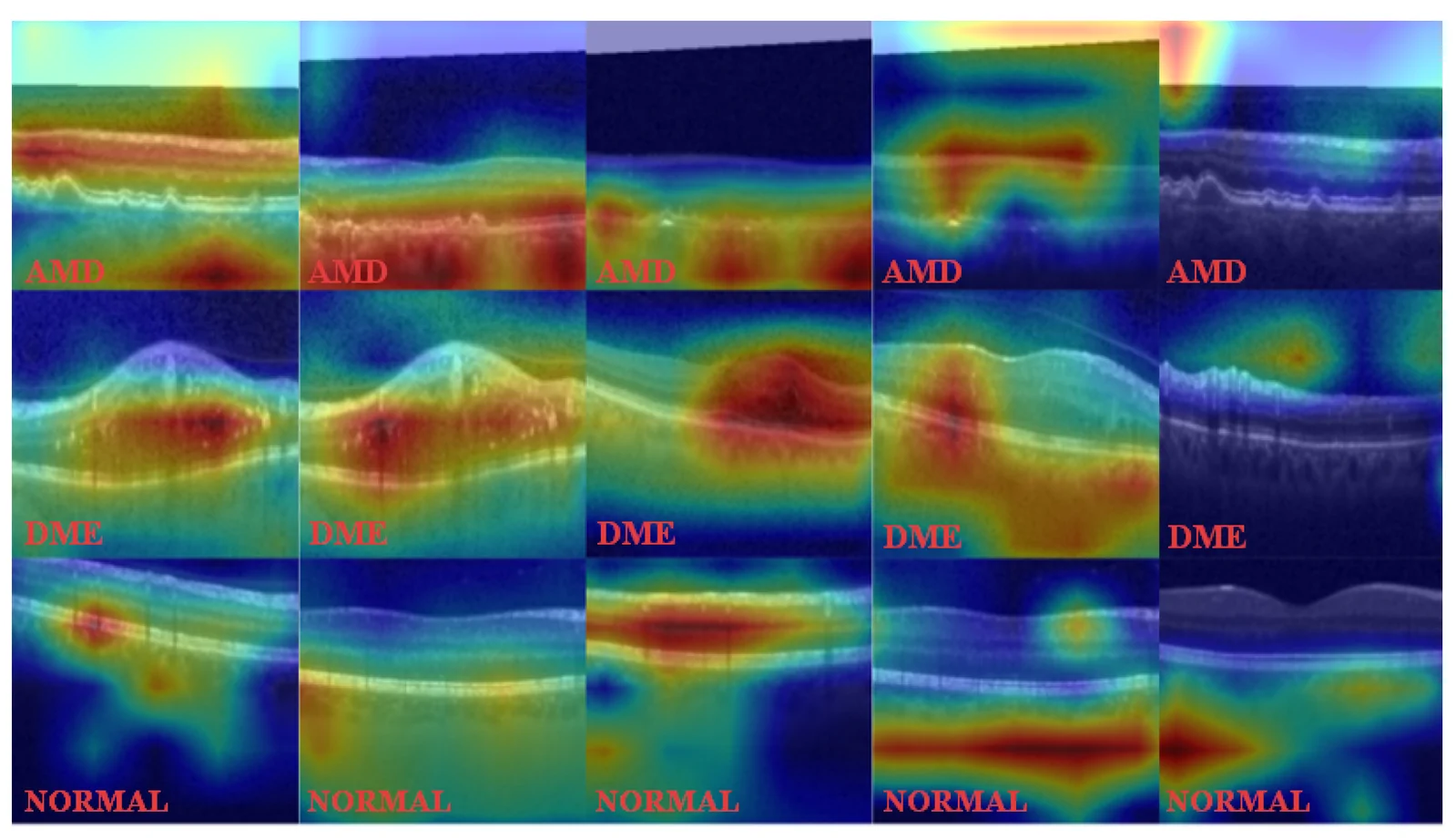
Grad-CAM heatmaps illustrating the influence of different hyperparameter configurations (*k* and β) on representative TMI samples across AMD, DME, and NORMAL categories. The columns correspond to the hyperparameter configurations: (1) k=32,β=0.005; (2) k=64,β=0.001; (3) k=128,β=0.001; (4) k=16,β=0.05; and (5) k=16,β=0.1. The colors in the Grad-CAM heatmaps indicate the relative importance of different image regions to the model’s prediction. The importance progressively decreases from red (highest importance) to yellow, yellow-green/green, blue, and finally dark blue (lowest importance).

As shown in Figure 8, we further compared the selected four hyperparameter configurations with the optimal configuration of our model, i.e., k=32,β=0.005. By directly comparing these four settings against the optimal one, it can be clearly observed that the optimal configuration k=32,β=0.005 yields significantly better visual localization and attention concentration than the other four configurations. Moreover, our observations reveal the specific impacts of *k* and β. For the neighborhood size *k*, overly small values lead to insufficient local samples for precise LID estimation, which tends to cause scattered attention. Conversely, excessively large values may introduce non-local samples from outside the target region, resulting in blurred attention localization in the heatmaps. Regarding the regularization weight β, values that are too low weaken the suppression of dimensional collapse in the feature space, while excessively high values may over-constrain the geometric structure, leading to undesirable attention shifts.

Overall, these Grad-CAM visualizations provide qualitative evidence that selecting moderate hyperparameter configurations (k=32,β=0.005) effectively encourages the model to focus on clinically relevant retinal structures while preserving feature expressiveness, particularly when the $L_{1}$ formulation is adopted.

## 5. Conclusions

This study presents an OCT-oriented adaptation of the original LDReg framework for self-supervised retinal OCT representation learning. By integrating local intrinsic dimensionality estimation and asymptotic Fisher-Rao regularization into a self-supervised learning pipeline, the proposed framework effectively preserves local anatomical and pathological variations while mitigating local dimensional degeneration in the representation space. Unlike the original LDReg framework developed for general self-supervised representation learning, this work demonstrates that local intrinsic dimensionality regularization can be successfully transferred to retinal OCT image analysis through OCT-specific framework design and comprehensive experimental validation.

The effectiveness of RetiLID was evaluated on the TMI, BOE, and CELL datasets using a linear evaluation protocol. The proposed method achieved classification accuracies of 94.35%, 92.48%, and 92.56% on the three datasets, respectively, and consistently performed favorably compared with the evaluated self-supervised learning approaches. The quantitative metrics, together with the ROC, PR, and confusion-matrix analyses, demonstrate that incorporating local dimensional information improves the discriminative quality of self-supervised OCT representations across datasets with different scales and disease categories.

The present study demonstrates the feasibility and effectiveness of adapting local intrinsic dimensionality regularization to self-supervised retinal OCT representation learning. Future work will further extend the proposed OCT-oriented framework in several directions. First, the framework will be evaluated on larger multi-center and multi-device retinal OCT datasets to further assess its robustness and generalization across different imaging devices, acquisition protocols, and clinical populations. Second, richer retinal anatomical priors, including retinal layer structures and lesion-aware information, will be incorporated into neighborhood construction and local intrinsic dimensionality regularization to better exploit OCT-specific structural characteristics. Third, interpretable learning techniques, such as Grad-CAM and local feature visualization, will be integrated to provide intuitive evidence of model decision-making and to better illustrate the influence of local geometric regularization on retinal disease classification. These future developments are expected to further improve the robustness, interpretability, and clinical applicability of the proposed framework for retinal OCT image analysis.

## Figures and Tables

**Table 1:** Semi-supervised evaluation settings and dataset splits.

Category	Dataset	Value
1% labels	BOE	∼22 images
	TMI	∼24 images
	CELL	∼1082 images
10% labels	BOE	∼224 images
	TMI	∼238 images
	CELL	∼10,824 images
Random seeds		1228, 1328, 1428
Report format		mean ± standard deviation
Dataset splits	BOE	3231 B-scans: Train 2248/Val 983
	TMI	3798 B-scans: Train 2382/Val 1026/Test 390
	CELL	109,309 B-scans: Train 108,242/Val 1067
Split level	BOE	Patient-level (no overlap)
	TMI	Patient-level (TMIdata_split_by_person)
	CELL	Image-level [3]
Official splits		Used for all three datasets
Test set isolation	TMI	Test set (390 images) excluded from training

**Table 2:** Self-supervised pre-training hyperparameters.

Hyperparameter	Value
Backbone	ResNet-50
Projector	3-layer FC (2048 → 1024 → 1024 → 1024), BatchNorm + ReLU
Optimizer	SGD
Initial learning rate	0.05×batch_size/256
Scheduler	CosineAnnealingLR (150 epochs, no warmup)
Weight decay	1 × 10^−4^
Momentum	0.9
Batch size	16
Augmentation	RandomResizedCrop (224, scale = 0.2–1.0), RandomHorizontalFlip, ColorJitter (0.4, 0.4, 0.4, 0.2), RandomGrayscale (p=0.2), GaussianBlur (σ=0.1–2.0, p=0.5)

**Table 3:** Linear evaluation settings.

Configuration	Value
Encoder status	Frozen
Trainable parameters	Only nn.Linear(2048, num_classes)
Optimizer	SGD (lr = 0.3, momentum = 0.9, weight_decay = 1 × 10^−4^, nesterov = True)
Scheduler	CosineAnnealingLR ($T_{\max}=100$ epochs)
SSL pre-training	All images in train/ (labels ignored)
Semi-supervised eval	Only sampled labeled subset used
Remaining unlabeled samples	Not used

**Table 4:** Comparison of linear evaluation and semi-supervised classification performance on the TMI dataset.

Method	Linear			Semi-Supervised 1%			Semi-Supervised 10%		
	Accuracy	Specificity	Sensitivity	Accuracy	Specificity	Sensitivity	Accuracy	Specificity	Sensitivity
SimCLR [20]	0.825±0.009	0.942±0.005	0.826±0.009	0.727±0.021	0.850±0.011	0.685±0.024	0.816±0.018	0.947±0.008	0.816±0.018
SimSiam [21]	0.853±0.008	0.916±0.004	0.856±0.008	0.704±0.018	0.840±0.010	0.696±0.019	0.824±0.015	0.918±0.009	0.845±0.013
BarlowTwins [22]	0.780±0.013	0.939±0.007	0.780±0.013	0.630±0.028	0.852±0.014	0.616±0.029	0.762±0.020	0.936±0.011	0.768±0.019
LFR [24]	0.670±0.024	0.835±0.012	0.670±0.024	0.515±0.034	0.758±0.019	0.515±0.034	0.638±0.026	0.819±0.013	0.638±0.026
AimV2 [26]	0.918±0.004	0.959±0.002	0.918±0.004	0.721±0.011	0.861±0.006	0.721±0.011	0.885±0.008	0.943±0.004	0.885±0.008
I-JEPA [25]	0.928±0.003	0.964±0.002	0.928±0.003	0.689±0.013	0.844±0.007	0.697±0.013	0.876±0.009	0.938±0.005	0.875±0.009
RetiLID	0.944±0.003	0.954±0.002	0.947±0.003	0.796±0.009	0.877±0.005	0.789±0.009	0.933±0.006	0.952±0.003	0.936±0.006

**Table 5:** Comparison of linear evaluation and semi-supervised classification performance on the BOE dataset.

Method	Linear			Semi-Supervised 1%			Semi-Supervised 10%		
	Accuracy	Specificity	Sensitivity	Accuracy	Specificity	Sensitivity	Accuracy	Specificity	Sensitivity
SimCLR [20]	0.903±0.006	0.951±0.003	0.903±0.006	0.618±0.031	0.809±0.015	0.618±0.031	0.849±0.036	0.925±0.018	0.849±0.036
SimSiam [21]	0.799±0.018	0.911±0.015	0.799±0.018	0.752±0.013	0.876±0.006	0.752±0.012	0.843±0.034	0.906±0.011	0.843±0.034
BarlowTwins [22]	0.905±0.014	0.953±0.008	0.905±0.014	0.688±0.037	0.844±0.018	0.688±0.037	0.805±0.038	0.902±0.019	0.805±0.038
LFR [24]	0.523±0.035	0.762±0.017	0.523±0.035	0.381±0.046	0.691±0.023	0.381±0.046	0.543±0.031	0.771±0.016	0.543±0.031
AimV2 [26]	0.927±0.003	0.963±0.001	0.927±0.003	0.747±0.013	0.873±0.006	0.747±0.013	0.826±0.008	0.913±0.012	0.826±0.008
I-JEPA [25]	0.849±0.006	0.932±0.003	0.849±0.006	0.789±0.011	0.901±0.006	0.789±0.011	0.867±0.012	0.941±0.007	0.867±0.012
RetiLID	0.925±0.006	0.954±0.002	0.943±0.003	0.835±0.006	0.906±0.005	0.854±0.002	0.906±0.005	0.950±0.006	0.933±0.003

**Table 6:** Comparison of linear evaluation and semi-supervised classification performance on the CELL dataset.

Method	Linear			Semi-Supervised 1%			Semi-Supervised 10%		
	Accuracy	Specificity	Sensitivity	Accuracy	Specificity	Sensitivity	Accuracy	Specificity	Sensitivity
SimCLR [20]	0.701±0.001	0.901±0.001	0.701±0.001	0.647±0.005	0.882±0.002	0.647±0.005	0.737±0.044	0.912±0.014	0.737±0.044
SimSiam [21]	0.696±0.035	0.898±0.011	0.696±0.035	0.563±0.008	0.854±0.003	0.563±0.008	0.666±0.003	0.889±0.001	0.666±0.003
BarlowTwins [22]	0.783±0.005	0.927±0.001	0.783±0.005	0.805±0.011	0.935±0.004	0.805±0.011	0.757±0.054	0.919±0.018	0.757±0.054
LFR [24]	0.720±0.019	0.907±0.006	0.720±0.019	0.771±0.016	0.924±0.005	0.771±0.016	0.903±0.010	0.968±0.003	0.903±0.010
AimV2 [26]	0.866±0.002	0.955±0.004	0.866±0.005	0.896±0.002	0.951±0.005	0.896±0.002	0.923±0.002	0.959±0.002	0.923±0.002
I-JEPA [25]	0.864±0.003	0.932±0.003	0.864±0.003	0.870±0.004	0.934±0.003	0.870±0.004	0.918±0.003	0.944±0.006	0.918±0.003
RetiLID	0.926±0.005	0.953±0.009	0.919±0.009	0.897±0.006	0.945±0.003	0.886±0.001	0.920±0.002	0.950±0.006	0.914±0.003

**Table 7:** Sensitivity analysis of hyperparameter *k* on the TMI, BOE, and CELL datasets. The bold values indicate the best performance obtained for each dataset under different hyperparameter settings.

*k*	TMI	BOE	CELL
16	92.10	89.73	90.97
32	**94.35**	90.88	**92.56**
64	93.72	91.64	91.88
128	94.28	**92.48**	90.71

**Table 8:** Sensitivity analysis of hyperparameter β on the TMI, BOE, and CELL datasets. The bold values indicate the best performance obtained for each dataset under different hyperparameter settings.

β	TMI	BOE	CELL
0.1	91.85	90.65	91.01
0.05	93.10	91.42	91.78
0.01	94.02	**92.48**	92.24
0.005	**94.35**	91.95	**92.56**
0.001	93.21	91.16	91.63
0.000005	91.92	89.98	90.75

**Table 9:** Component ablation study on the TMI dataset. The bold values indicate the best performance obtained for each dataset under different hyperparameter settings. The checkmark (✓) indicates that the corresponding component is included in the configuration.

Configuration	LID	Fisher-Rao	100%	10%	1%
Baseline SSL			88.76±0.20	84.96±0.75	77.19±0.57
+LID	✓		89.90±0.17	85.15±0.99	78.27±1.76
+Fisher-Rao $\left(L_{1}\right)$	✓	$L_{1}$	90.25±0.08	86.03±1.06	78.75±1.77
+Fisher-Rao $\left(L_{2}\right)$	✓	$L_{2}$	89.99±0.28	85.48±1.06	78.69±1.77

**Table 10:** Comparison between the original LDReg framework [13] and the proposed RetiLID framework under the same evaluation protocol.

Dataset	Method	100% Labels	10% Labels	1% Labels
BOE	Original LDReg [13]	81.5%	79.2%	76.4%
	Proposed RetiLID	92.5%	90.6%	83.5%
TMI	Original LDReg [13]	83.4%	81.8%	77.2%
	Proposed RetiLID	94.4%	93.3%	79.6%
CELL	Original LDReg [13]	87.2%	85.9%	84.7%
	Proposed RetiLID	92.6%	92.0%	89.7%

## Data Availability

The datasets used in this study are publicly available. The TMI dataset is available at https://drive.google.com/file/d/1Rv82F7CjPveyONdy1YbRHh05emCb6_Eu/view (accessed on 20 August 2026). The BOE dataset is available at http://people.duke.edu/~sf59/ (accessed on 20 August 2026). The CELL dataset is available at https://data.mendeley.com/datasets/rscbjbr9sj/3 (accessed on 20 August 2026).

## Abbreviations

The following abbreviations are used in this manuscript:

OCT	Optical coherence tomography
AMD	Age-related macular degeneration
DME	Diabetic macular edema
CNV	Choroidal neovascularization
SimCLR	Simple Contrastive Learning of Visual Representations
SimSiam	Simple Siamese Representation Learning
LDReg	Local Dimensionality Regularization
LID	Local Intrinsic Dimension
ResNet	Residual Neural Network
LFR	Local Feature Reconstruction
I-JEPA	Image-based Joint-Embedding Predictive Architecture
AIM V2	Autoregressive Image Model v2
SSL	Self-Supervised Learning
AFR	Asymptotic Fisher–Rao distance
CNN	Convolutional Neural Network
ROC	Receiver Operating Characteristic
PR	Precision–Recall
